# The coevolution of play and the cortico-cerebellar system in primates

**DOI:** 10.1007/s10329-017-0615-x

**Published:** 2017-06-15

**Authors:** Max Kerney, Jeroen B. Smaers, P. Thomas Schoenemann, Jacob C. Dunn

**Affiliations:** 1 Division of Biological Anthropology, University of Cambridge, Pembroke Street, Cambridge, CB2 3QG UK; 2 Department of Anthropology, Stony Brook University, Stony Brook, NY USA; 3 Department of Anthropology and Cognitive Science Program, Indiana University, Bloomington, IN USA; 4 Stone Age Institute, Gosport, IN USA; 5 Animal and Environment Research Group, Anglia Ruskin University, Cambridge, UK

**Keywords:** Phylogenetic comparative methods, Brain evolution, Prefrontal cortex, Cognition, Cortical association areas

## Abstract

Primates are some of the most playful animals in the natural world, yet the reason for this remains unclear. One hypothesis posits that primates are so playful because playful activity functions to help develop the sophisticated cognitive and behavioural abilities that they are also renowned for. If this hypothesis were true, then play might be expected to have coevolved with the neural substrates underlying these abilities in primates. Here, we tested this prediction by conducting phylogenetic comparative analyses to determine whether play has coevolved with the cortico-cerebellar system, a neural system known to be involved in complex cognition and the production of complex behaviour. We used phylogenetic generalised least squares analyses to compare the relative volume of the largest constituent parts of the primate cortico-cerebellar system (prefrontal cortex, non-prefrontal heteromodal cortical association areas, and posterior cerebellar hemispheres) to the mean percentage of time budget spent in play by a sample of primate species. Using a second categorical data set on play, we also used phylogenetic analysis of covariance to test for significant differences in the volume of the components of the cortico-cerebellar system among primate species exhibiting one of three different levels of adult-adult social play. Our results suggest that, in general, a positive association exists between the amount of play exhibited and the relative size of the main components of the cortico-cerebellar system in our sample of primate species. Although the explanatory power of this study is limited by the correlational nature of its analyses and by the quantity and quality of the data currently available, this finding nevertheless lends support to the hypothesis that play functions to aid the development of cognitive and behavioural abilities in primates.

**Electronic supplementary material:**

The online version of this article (doi:10.1007/s10329-017-0615-x) contains supplementary material, which is available to authorized users.

## Introduction

Primates are exceptionally playful. Not only do primates engage in all of the major forms of play (locomotor, object, and social), but they also tend to spend more time playing than the members of practically any other taxonomic group (Burghardt 2005; Pellis et al. 2015). But why are primates so playful? One hypothesis posits that the distinctive quantity and quality of primate play are the result of a functional link between play and another distinctive characteristic of primates—their sophisticated cognitive and behavioural abilities. These include the capacity for extractive foraging, tool use, behavioural innovation, and complex sociality (e.g. Reader et al. 2011). It has been a long-standing idea that the repetitive ‘experimental’ activity characteristic of play (Burghardt 2005) may function to train and maintain such abilities. For example, Groos (1898) believed that play allowed young animals to practise and refine the complex behavioural patterns that they would require in adulthood, while Carr (1902) thought that play developed social skills. More recently, Byers and Walker (1995) have suggested that play may function to develop the motor skills necessary for the execution of complex behaviours, while Pellis and Pellis (2009) and Panksepp and Biven (2012) have argued that play can serve to develop socioemotional intelligence.

If play does function to aid the development of cognitive and behavioural skills, and if this is the reason for the high incidence of play in primates, then one might expect to observe: (1) a positive association between the incidence of play and the incidence of complex behaviours across primate species, and (2) a coevolutionary relationship between play and the neural substrates underlying complex cognition and behaviour. The first prediction has been substantiated by Montgomery (2014), who found a significant positive association between play and the frequency of extractive foraging, tool use, behavioural innovation, and tactical deception across 11 primate species. The second prediction has been investigated in studies of several gross-anatomical brain structures, including the neocortex (Lewis 2000), cerebellum (Lewis and Barton 2004), amygdala and hypothalamus (Lewis and Barton 2006), and striatum (Graham 2011). However, focussing on specific gross-anatomical brain structures might not be the best test of this second prediction for two fundamental reasons. First, it has become increasingly recognised that complex behaviours such as tool use and sociality are not the product of single brain structures, but instead emerge from the activity of distributed neural systems (e.g. Barton 2012). Second, the constituent areas of distributed neural systems do not comprise gross-anatomical structures such as neocortex or cerebellum, but rather more localized functionally specific areas within such gross-anatomical structures (Ramnani 2006). This point is underlined by the long-established fact that cross-species changes in overall neocortex size mainly relate to changes in heteromodal association areas, whereas changes in primary sensory cortices remain relatively stable (Diamond and Hall 1969; Passingham 1975; Buckner and Krienen 2013; Smaers et al. 2017). Likewise for the posterior and anterior cerebellum (MacLeod et al. 2003; Smaers 2014). In other words, gross anatomical structures like neocortex and cerebellum are not functionally homogeneous, do not scale in a homogeneous manner, and are therefore poor neurobiological proxies of behavioural capacity. A better test of a putative relationship between play and the neural substrate underlying cognitive and behavioural abilities is thus to test whether play in primates has coevolved with functionally relevant distributed association networks in the brain.

In this study we explore whether play has coevolved with the cortico-cerebellar system, a neural system known to underlie complex cognition and the production of complex behaviour in primates. The cortico-cerebellar (or ‘cerebro-cerebellar’) system is one of the largest projection systems in the primate brain; its principal constituent areas include heteromodal cortical association areas of the neocortex (e.g. prefrontal, premotor and parietal cortex) and posterior hemispheric lobules of the cerebellum (Schmahmann and Pandya 1997; Ramnani 2006; Glickstein et al. 2011). These areas of the neocortex and cerebellum are anatomically linked (Kelly and Strick 2003) and work in alliance to facilitate complex cognition and the production of complex behaviour (Koziol et al. 2014). The manner in which the cortico-cerebellar system supports complex behaviour can be described by means of the hierarchical mapping of information in the brain. Sensory information ascends from primary sensory areas to temporo-parietal association areas where it is integrated and transformed into mental representations. Aggregates of mental representations (i.e. mental models) can be understood as small-scale models of reality that are used to reason, to explain current events, and to anticipate future events (Johnson-Laird 1983). The prefrontal cortex performs executive control over these mental models, thereby exerting conscious control over thoughts and actions in accordance with internal goals (Miller and Cohen 2001). The posterior cerebellar hemispheres integrate mental models with external stimuli and self-generated responses into internal models of sequences of thoughts and actions (Brindley 1964; Schmahmann 1997, 2000; Ito 2006; Ramnani 2006). Such internal models allow for a smooth context-dependent execution of sequences of thoughts and actions (Koziol et al. 2014). Drawing on prefrontal executive control, the cerebellum can update these internal models if misalignment is detected between the internal model and self-generated goals (Ito 2006, 2008). The cerebellum’s capacity to update internal models forms the basis of cumulative learning and hereby helps optimize the performance of behaviours according to context. Previous studies have demonstrated that the principal constituent areas of the cortico-cerebellar system (prefrontal cortex, non-prefrontal heteromodal cortical association areas, and the posterior hemispheric lobules of the cerebellum) have significantly expanded in lockstep with the grade shifts in cognitive and behavioural complexity observed between monkeys, (great) apes, and humans (MacLeod et al. 2003; Smaers et al. 2011, 2013; Smaers 2014; Passingham and Smaers 2014; Passingham et al. 2017; Smaers et al. 2017). If play functions to develop the cognitive and behavioural abilities of primates then one might expect a coevolutionary relationship to exist across species between play and the principal constituent areas of the cortico-cerebellar system.

## Methods

We compiled two types of data on primate play. The first was data on the mean percentage of time budget (across age and sex classes) allocated to play (of all types) by primate species. A set of such data has previously been compiled and used in comparative studies of primate play (e.g. Graham 2011; Montgomery 2014). However, in reviewing the primary literature underpinning this data set it was unclear for some species how the species mean values had been calculated from the primary data contained in that literature. We therefore returned to the primary literature to compile our own data set, adding additional data from new sources where possible. A detailed description of the procedure that we used to collect time budget data from the literature is given in Online Resource 1. We were able to compile data on the percentage of the time budget spent in play for 8 primate species for which data on the cortico-cerebellar system were also available (Online Resource 2).

The second data set consisted of ordinal data on the frequency of adult-adult social play. These data were taken from Iwaniuk et al. (2001), who used qualitative descriptions of the frequency of adult-adult social play in primate species to assign species a score between 0 and 4, with 0 denoting that such play has not been observed or is rare in that species and 4 that it is very common. Of the species included in Iwaniuk et al.’s (2001) data set, data on the cortico-cerebellar system were available for 13 of them, although not for any species with a score of 4, and for only one species with a score of 2. As a result, these play frequency categories were excluded from our data set, leaving us with data for 12 primate species assigned to one of three categories in which adult-adult social play was reported as either unobserved, infrequent, or common in that species (Online Resource 2).

Both of the data sets on primate play compiled for this study have their advantages and limitations. The data set collected on the mean percentage of time budget allocated to play by species provides relatively fine-grained data on the overall playfulness of different species. However, it lacks data on the percentage of time spent in play by specific age and sex classes, and on the percentage of time spent in specific types of play (e.g. locomotor, object, or social). This is due to a shortage of such data in the primary literature. This data set is also limited by the problems inherent to the amalgamation of quantitative data from diverse primary sources to create single mean values to represent species. Each of these primary sources can vary in terms of the data collection methods and the definitions of play that they use, their sample sizes and sample compositions, and focus on either captive or wild subjects, all of which can impact the final mean values that are derived from them. The data set collected on the frequency of adult-adult social play has the advantages of providing information on play in a specific age class and on a specific type of play, and, since the data originate from a single source, the methods used to generate the data were consistent. However, the highly specific nature of the type of play being considered, combined with the relatively simple rating scale that is used, means that this data set may not provide the most accurate representation of the overall playfulness of each species. Both data sets are also limited by their small sample sizes, which are constrained by the amount of data currently available in the literature on both play and on the cortico-cerebellar system in primates. Used together, however, we believe that these two data sets are complementary and mutually reinforcing, and are capable of providing independent insight into the hypothesis under consideration.

We obtained volumetric data on prefrontal cortex, non-prefrontal heteromodal cortical association areas, and posterior cerebellar hemispheres from Smaers et al. (2010, 2011, 2013). In addition, we obtained data on the volumes of the primary visual cortex (striate cortex grey matter) and the medial anterior cerebellum (Frahm et al. 1983; de Sousa et al. 2010; Smaers et al. 2011, 2013) (Online Resource 2). These two structures are not associated with the production of complex behaviour—being involved primarily in visual processing (visual cortex) and proprioception, autonomic functions and basic motor control (medial anterior cerebellum)—and so can act as control structures. All brain data were log transformed before analysis. Data on the relative size of brain areas were derived from allometric residuals generated by phylogenetic generalised least squares (PGLS) regressions of the data on each structure against data on an appropriate comparative structure. For a detailed description of this procedure see Online Resource 3.

The first set of phylogenetic comparative analyses consisted of PGLS analyses incorporating a consensus tree from the 10kTrees project (Arnold et al. 2010) to determine the cross-species relationship between the mean percentage of time budget spent in play and the relative sizes of: (1) the individual principal constituent areas of the cortico-cerebellar system (prefrontal cortex grey matter, non-prefrontal heteromodal cortical association area grey matter, posterior cerebellum); (2) the summed volume of the principal constituent areas of the cortico-cerebellar system (prefrontal cortex grey matter and posterior cerebellum, non-prefrontal heteromodal cortical association area grey matter and posterior cerebellum); and (3) the control structures (primary visual cortex, medial anterior cerebellum). The *P*-values obtained from these analyses were adjusted for multiple comparisons using the Benjamini and Hochberg correction (1995).

The second set of analyses consisted of phylogenetic analysis of covariance (pANCOVA) analyses (Smaers and Rohlf 2016) to determine whether significant differences in the relative sizes of the neural structures exist among the species of the different play frequency categories in the adult-adult social play data set. All of our analyses were carried out within the R software environment (R Core Team 2014).

## Results

**Fig. 1:**
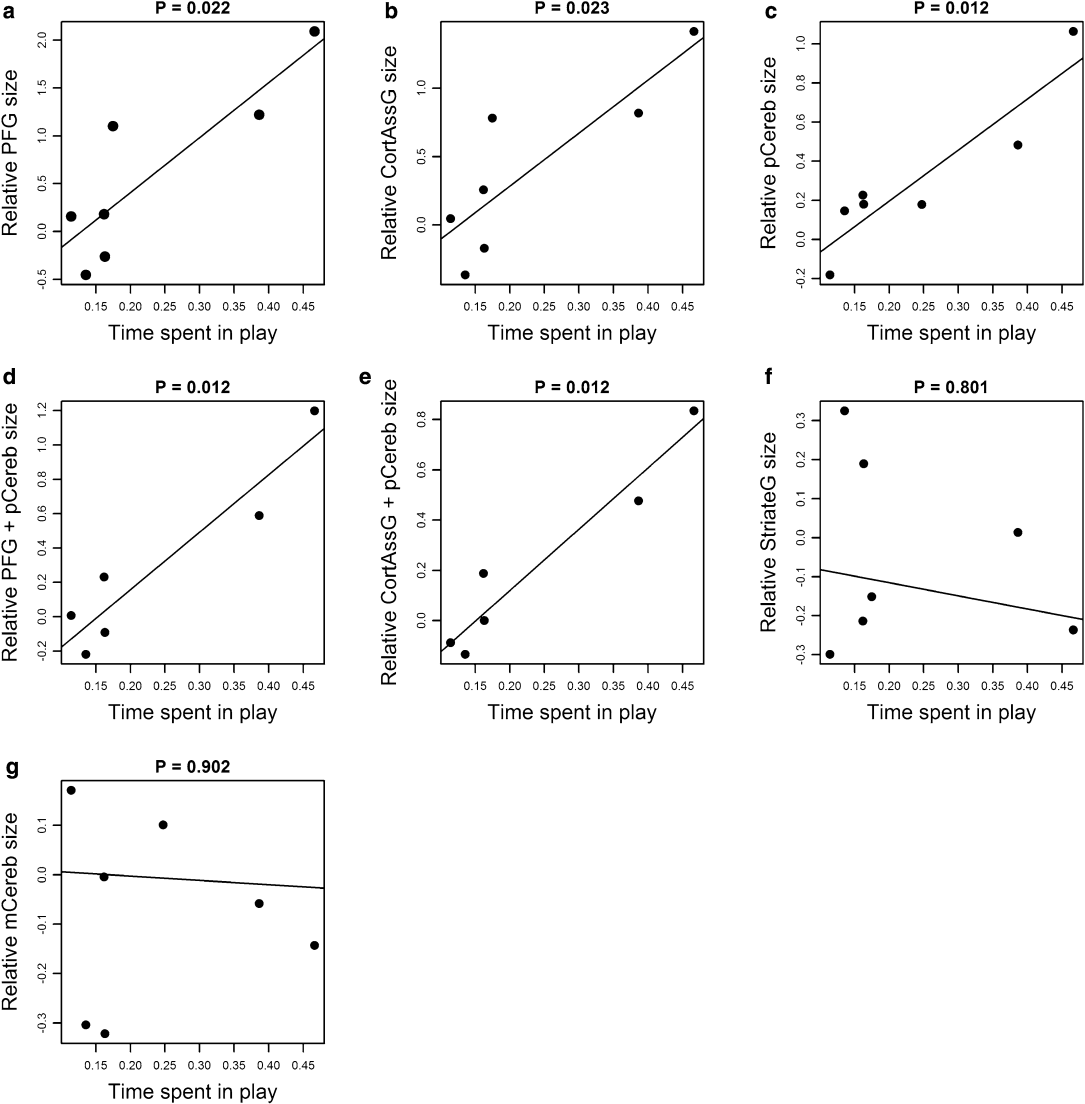
Plots of the phylogenetic generalised least squares regressions of mean percentage of time budget spent in play (arcsine transformed) against residual data representing the relative sizes of: **a** prefrontal cortex grey matter (*PFG*), **b** non-prefrontal heteromodal cortical association area grey matter (*CortAssG*), **c** the posterior cerebellum (*pCereb*), **d** prefrontal cortex grey matter and posterior cerebellum (*PFG* + *pCereb*), **e** non-prefrontal heteromodal cortical association area grey matter and posterior cerebellum (*CortAssG* + *pCereb*), **f** the primary visual cortex (*StriateG*), **g** the medial anterior cerebellum (*mCereb*)

We found significant, positive associations between the mean percentage of time budget spent in play and the relative sizes of: prefrontal cortex grey matter (*R* ^2^ = 0.743, *P* = 0.022, *λ* = 0; Fig. 1a), non-prefrontal heteromodal cortical association area grey matter (*R* ^2^ = 0.717, *P* = 0.023, *λ* = 0; Fig. 1b), posterior cerebellum (*R* ^2^ = 0.835, *P* = 0.012, *λ* = 0; Fig. 1c), prefrontal cortex grey matter and posterior cerebellum (*R* ^2^ = 0.886, *P* = 0.012, *λ* = 0; Fig. 1d), and non-prefrontal heteromodal cortical association area grey matter and posterior cerebellum (*R* ^2^ = 0.930, *P* = 0.012, *λ* = 0; Fig. 1e). No significant associations were found between these play data and the relative sizes of the control structures, i.e. the primary visual cortex (*R* ^2^ = 0.035, *P* = 0.801, *λ* = 0.754; Fig. 1f) and the medial anterior cerebellum (*R* ^2^ = 0.003, *P* = 0.902, *λ* = 1; Fig. 1g).

In the second set of analyses we found significant differences between the most playful and the least playful species in the relative sizes of prefrontal cortex grey matter and non-prefrontal heteromodal cortical association area grey matter (Table 1). To establish the magnitude of these differences, we determined what the relative sizes of the brain structures would be predicted to be in species in the most playful category if those species were on the same regression line as those in the least playful category, and then calculated a ratio of the observed to predicted values. This ratio (the ‘corticalisation coefficient’; Table 1) provides an indication of how many times larger the brain structures of the most playful species are compared to those of the least playful species. For example, the volume of prefrontal cortex grey matter was calculated to be between 1.5 and 9 times greater in species in which play is common compared to the volume that would be predicted if play was unobserved in those species, demonstrating that the difference between groups is not only statistically significant but also likely to be biologically meaningful. We found no differences in the relative sizes of any other structures between the species of the different play frequency categories. The failure to detect significant differences between groups in the relative sizes of the posterior cerebellum and the summed components of the cortico-cerebellar system may be due to the highly specific measure of play used in these analyses.

**Table 1:**
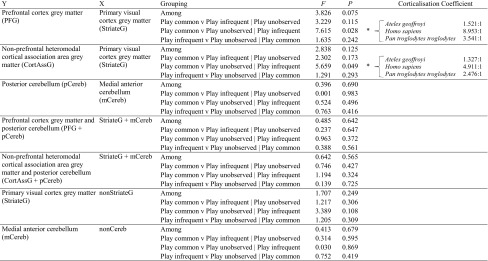
The results of the phylogenetic analysis of covariance analyses

“Group X v Group Y” denotes the groups that are being compared for a significant difference in brain structure size; “ | Group Z” denotes the control group for that comparison. In cases where there is a significant difference between groups the Corticalisation Coefficient provides an indication of how many times larger the brain structure is in the species of the higher play group compared to the lower play group, being a ratio of the observed size of the brain structure in the species of the higher play group to the size that would be predicted for those species if they belonged to the lower play group

## Discussion

Our results suggest that in our data set there is, in general, a positive association between play and the relative size of the components of the cortico-cerebellar system, a major projection system in the primate brain that underlies complex skills such as extractive foraging, tool use, and sociality (e.g. Ramnani 2006; Koziol et al. 2014; Smaers 2014). Although the limitations of the data used in this study restrict our ability to generalise, the results do nevertheless lend support to the hypothesis that the high level of play observed in primates is due to a functional link between play and the development of cognitive and behavioural abilities. More specifically, our results support the prediction of the hypothesis that play should be associated with the neural substrate of those abilities. This prediction is further supported by the finding that play seems to be associated specifically with the neural substrate of those abilities and not with other unrelated neural structures.

As the analyses conducted in this study were purely correlational it is important to bear in mind the possibility of alternative explanations for the results. For example, play may be associated with the cortico-cerebellar system not because play functions to develop the cognitive and behavioural skills that that system helps to produce, but because play—as a complex behaviour—is itself produced by that system. We consider that this is unlikely to be the case, however, since experimental studies have shown that while cortical systems can be involved in the modulation of play behaviour, they are not responsible for the initial production of play behaviour or for the absolute amount of play exhibited, with animals being capable of extensive play even in the absence of a cortex (Pellis and Pellis 2009, 2016). Another more viable alternative explanation for the results is that play and the cortico-cerebellar system may have coevolved because of a common association with particular ecological or life history variables.

Although this study does not have the requisite scope or power for us to be able to rule out such alternative explanations, its findings do contribute to a growing pattern of evidence that suggests that a functional relationship does exist between play and the development of the cognitive and behavioural abilities of primates (Lewis 2000; Lewis and Barton 2004, 2006; Graham 2011; Montgomery 2014). This pattern of evidence seems suggestive enough to warrant further investigation of the hypothesis. Essential to any further investigations will be the development of a much more extensive, detailed, and reliable database of primate play and brain data in order to give future studies a more satisfactory level of explanatory power than is currently possible. These future studies should include not only larger-scale phylogenetic comparative studies to validate the results found in this and previous studies, but also experimental studies to directly test the causal relationships that are suggested to be involved. Through such efforts we may be able to substantially advance our understanding of the extraordinary playfulness of primate species, including that of our own.

## Electronic supplementary material

Below is the link to the electronic supplementary material.

Descriptions of the procedure used to collect time budget data from the literature and of how the mean percentage of time budget data for each species were derived (XLS 49 kb)

The play and brain data used in the study (XLSX 15 kb)

Details of the procedure used to calculate the relative sizes of the brain structures (PDF 88 kb)
