# Supplementary material for: The coevolution of play and the cortico-cerebellar system in primates
Source: Primates. 2017 Jun 15;58(4):485–91. doi: 10.1007/s10329-017-0615-x (PMC5622916; doi:10.1007/s10329-017-0615-x)
Supplement: Supplementary file 3 — Details of the procedure used to calculate the relative sizes of the brain structures (PDF 88 kb) [file 10329_2017_615_MOESM3_ESM.pdf]

ONLINE RESOURCE 3

Paper title: The Coevolution of Play and the Cortico-Cerebellar System in Primates
Journal: Primates
Authors: Max Kerney*, Jeroen B. Smaers, P. Thomas Schoenemann, Jacob C. Dunn
(*corresponding author)
Affiliation of corresponding author: Division of Biological Anthropology, University of Cambridge, Cambridge, UK
Email address of corresponding author: mkerney@cantab.net

Details of the procedure used to calculate the relative sizes of the brain structures examined in the study.

Data on the relative size of the brain structures examined in this study were derived from allometric residuals generated by phylogenetic generalised least squares (PGLS) regressions of the data on each structure against data on an appropriate comparative structure.

In such a procedure it is crucial that the choice of appropriate comparative structures is made based on the functional and neuroanatomical realities of neural information processing. It has long been established that information processing in the brain occurs in a hierarchical manner. Stimuli are initially mapped onto primary sensory areas, and information subsequently projects to higher order association areas (Kaas 1997). Throughout this process perception and interpretation of stimuli becomes increasingly complex (Desimone and Schein 1987; Miller and Cohen 2001). Therefore, when seeking to determine the relative size of neural structures involved in complex cognitive processing, an approach that adequately accounts for the functional and neuroanatomical underpinnings of neural information processing is to compare the size of these structures which are at the top of the hierarchy of information processing to the size of those at the beginning of the hierarchy (primary sensory areas) (Passingham and Smaers 2014). This approach effectively compares the amount of complex information processing relative to the amount of sensory input. For

the assessment of cortical areas, such a comparison would involve comparing the size of cortical association areas (e.g., prefrontal cortex and other heteromodal association areas) relative to the size of primary sensory areas (e.g., primary visual cortex). For the assessment of cerebellar areas, this comparison would involve comparing the size of the posterior cerebellum relative to that of the medial anterior cerebellum (e.g., MacLeod et al. 2003). This approach is preferable to the more traditional procedure used to determine the relative size of brain structures, which compares the size of brain structures relative to the size of the rest of the brain. This more traditional procedure underestimates changes in neural systems (Passingham and Smaers 2014) and erroneously assumes that neural information processing is isolated in particular regions, thereby ignoring the well-established hierarchical nature of neural information processing.

In line with the above considerations, in this study the PGLS regressions used to generate the allometric residuals which indicate the relative size of each structure compared the volume of the prefrontal cortex (PFG) and the volume of other heteromodal cortical association areas (CortAssG) to the volume of primary sensory areas (i.e., primary visual cortex, StriateG), and the volume of posterior cerebellum (pCereb) to the volume of the medial anterior cerebellum (mCereb). The summed volumes of the principal constituent areas of the cortico-cerebellar system (PFG + pCereb, CortAssG + pCereb) were compared to the summed volume of the primary sensory areas and medial anterior cerebellum (StriateG + mCereb). The volumes of the control structures – primary visual cortex (StriateG) and medial anterior cerebellum (mCereb) – were compared to the volumes of the rest of neocortex grey matter (nonStriateG) and the rest of the brain (nonCereb) respectively (See Online Resource 2).

In order to maximise the accuracy of the residual data generated, these PGLS regressions used the largest sample of species for which brain data were available (N=19),

regardless of whether play data were available for these species or not (see Online Resource 2).

Prior to extracting the allometric residuals from such regression analyses, it is standard procedure to evaluate whether slopes and intercepts are homogenous among all groups in the sample being analysed (Sokal and Rohlf 2012). Significant differences in slopes or intercepts among groups in a sample indicate that not all groups are part of the same allometry, but rather that the sample is characterized by multiple grades (where each group is characterized by its own allometry) (Smaers and Rohlf 2016). When calculating the relative sizes of the brain structures in this study, we first established for each analysis whether grade shifts occurred among the humans, great apes, and other primates in the sample. If a grade shift was found to exist, then the allometric residuals were taken based on the allometry of the non-great ape portion of the sample. This procedure is justified because when residuals are taken from a one grade allometry when a multi-grade allometry is found to provide a better fit, then residuals do not accurately reflect the true relationship between size and form in the sample. Moreover, the nature of the phylogenetic relationship between non-great ape primates and great apes (great apes are embedded within the earlier radiation of monkeys) demonstrates that whatever trend is prevalent across monkeys is the ancestral condition of great apes. Therefore, the non-great ape sample is regarded as the most valid baseline against which to compare distinct great ape and human grades.

References

Desimone R, Schein SJ (1987) Visual properties of neurons in area V4 of the macaque: sensitivity to stimulus form. J Neurophysiol 57:835–868.

Kaas JH (1997) Topographic maps are fundamental to sensory processing. Brain Res Bull 44:107–112.

MacLeod CE, Zilles K, Schleicher A, et al (2003) Expansion of the neocerebellum in Hominoidea. J Hum Evol 44:401–429.

Miller EK, Cohen JD (2001) An integrative theory of prefrontal cortex function. Rev Neurosci 24:167–202.

Passingham RE, Smaers JB (2014) Is the Prefrontal Cortex Especially Enlarged in the Human Brain? Allometric Relations and Remapping Factors. Brain Behav Evol 84:156–66.

Smaers JB, Rohlf F (2016) Testing species’ deviations from allometric predictions using the phylogenetic regression. Evolution (N Y) 70:1145–9.

Sokal R, Rohlf F (2012) Biometry, 4th edn. W.H. Freeman and Company, New York
